# Oligonucleotide nanoassemblies with allyl bromide scaffold-based small molecules

**DOI:** 10.1186/s11671-023-03846-0

**Published:** 2023-06-03

**Authors:** Sk Jahir Abbas, Sabina Yesmin, Fangfang Xia, Sk Imran Ali, Zeyu Xiao, Weihong Tan

**Affiliations:** 1 Institute of Molecular Medicine, Renji Hospital, Shanghai Jiao Tong University School of Medicine, Shanghai, 200240 China; 2 Department of Physics, National Dong Hwa University, Hualien, 97401 Taiwan; 3 Department of Chemistry, University of Kalyani, Kalyani, West Bengal India; 4 Department of Pharmacology and Chemical Biology, Shanghai Jiao Tong University School of Medicine, Shanghai, 200025 China; 5 Institute of Basic Medicine and Cancer (IBMC), Chinese Academy of Sciences, Hangzhou, 310022 Zhejiang China; 6 Molecular Science and Biomedicine Laboratory (MBL), Aptamer Engineering Centre of Hunan Province, Hunan University, Changsha, 410082 Hunan China

## Abstract

The development of oligonucleotide nanoassemblies with small molecules has shown great potential in bio-medical applications. However, the interaction of negatively charged oligonucleotides with halogenated small molecules represents a scientific challenge. Here, we introduced a distinct allyl bromide halogenated scaffold, which exhibits specific interaction with adenine nucleic bases of the oligonucleotides, thus leading to the formation of self-assembled nanostructures.

**Graphical abstract:**

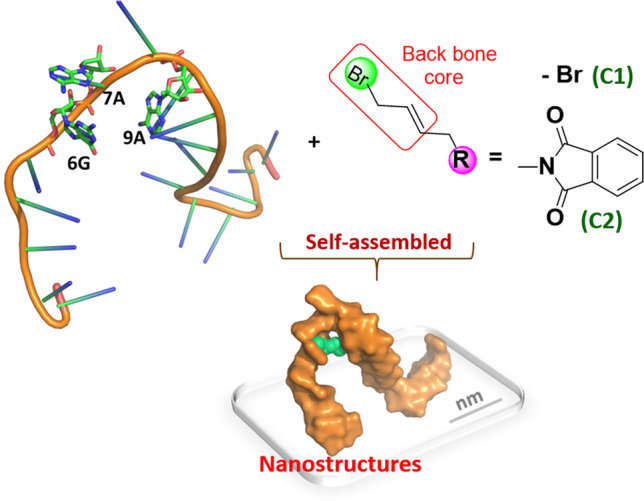

**Supplementary Information:**

The online version contains supplementary material available at 10.1186/s11671-023-03846-0.

## Introduction

**Fig. 1:**
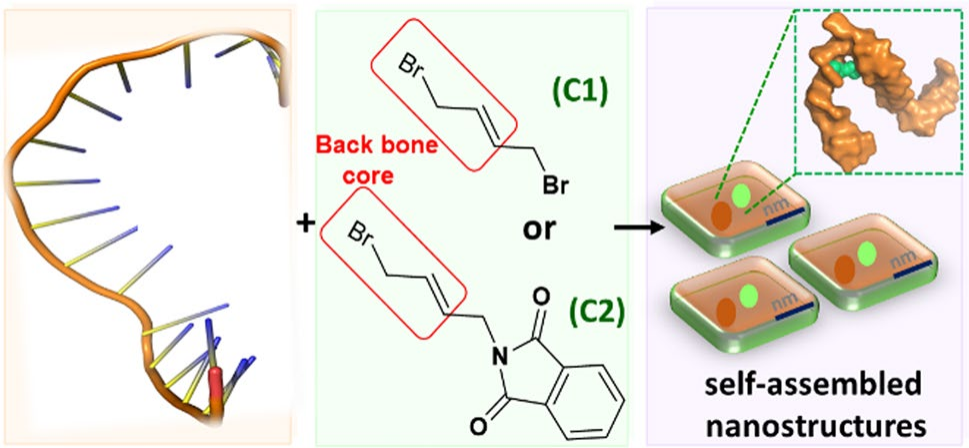
Schematic representation of self-assembled nanostructures between anionic small molecules and oligonucleotides

Oligonucleotide (ONs) nanoassemblies by interacting with smaller molecules have shown great potential for biomedical applications. Due to their negatively charged properties, oligonucleotides have been traditionally considered as binding agents for cationic small molecules, [1–4] with few reports of their ability to bind with anionic small molecules^5^. However, many anionic small molecules, including chloramphenicol (rr0061) and blasicidin S (rr0045) [5–7], have demonstrating medical promise in drug delivery, tumor targeting, and as antibiotic drugs. The formation of anionic nanoassemblies using small molecules is expected to solve the problems of cytotoxicity and rapid clearance by immune cells associated with cationic nanoassemblies [8]. The development of anionic nanoassemblies of oligonucleotides with halogenated small molecules represents a scientific challenge with great demand. In the present study, we identified a class of halogenated small molecules containing allyl bromide unit, which can bind to specific bases of oligonucleotides to form nanoassemblies (Fig. 1).

## Experimental details

### Synthesis and characterized of small molecules (C2)

The small molecule C2 synthesized based on a published procedure [9]. The condensation reaction between trans 1,4 dibromo-2-butene (1.06 g, 5 mmol, 2.00 equiv) and potassium phthalimide (463 mg, 2.50 mmol, 1.00 equiv), under anhydrous DMF (3 mL) and refluxing condition followed by purified with column chromatography 40% ethyl acetate/hexane desired products in 75% yields. The ^1^H NMR data match the literature [10]. ^1^H NMR (400 MHz, CDCl_3_) 7.85 (m, 2H), 7.73–7.72 (d, 2H), 5.98–5.91 (dtt, J = 16.2, 7.1, 1.2 Hz, 1H), 5.86–5.80 (dtt, J = 15.2, 6.0, 0.8 Hz, 1H), 4.31–4.29 (ddd, J = 6.0, 1.3, 0.7 Hz, 2H), 3.91–3.89 (dq, J = 7.3, 0.8 Hz, 2H); ^13^C NMR (100 MHz, CDCl_3_) 167.7, 134.0, 132.0, 129.9, 128.3, 123.3, 38.6, 31.2.

### Self-assembly of ONs nanoparticles

2 µM of ONs mixed with 25 mM of small molecules (dissolve in acidic methanol) and 20 mM Tris–HCl buffer (pH 6.2) contain total volume 20 µl followed by kept in 45 min at 42 °C. Subsequently, the unreacted ONs and small molecules were removed by dialysis in water for 2 days best on early reports [3].

### Polyacrylamide gel electrophoresis (PAGE) assay

PAGE performed to evaluate self-assembly ONs nanoparticles. 5 μL samples were loaded onto 5% polyacrylamide gels with 1 μL 6 × loading buffer under room temperature followed by electrophoresis at 100 V for 60 min and gels stained through GelRed. The results was analyzed with Bio-Rad ChemiDoc™ Touch Imaging System.

### Molecular dynamics simulations

The molecular AMBER force field (GAFF) parameters created through Antechamber implemented in AmberTools [11]. The 3D structural of ONs was projected using the in silico protocol [12, 13]. The unbiased molecular dynamics (MD) simulations carried out to understand the model structural of ONs bound to the small molecules. The experiment performed between two molecules (at 20 Å apart from each other) without any artificial force and placed in center of a truncated octahedral water box and 150 mM NaCl with a size of 64 × 64 × 64 Å^3^ and balanced the counter charge sodium ions use in the complexes MD simulations through AMBER ff99bsc-OL15 force field [14, 15] and general AMBER force field (GAFF) [11, 16] with Amber16 code [17, 18] scheduled in-time GPU clusters. On the other hand Åqvist potential [19] and TIP3P model [20] for the ions and water molecules, respectively. For the noncovalent interactions cutoff value was 10 Å and electrostatic interactions measured through Particle-Mesh Ewald (PME) method [21]. The covalent bonds were forced by SHAKE algorithm [22] and energy minimization achieved with steepest descent method [23].

### Binding free energy calculations

To calculated binding free energy between two molecules almost, 250 set of snapshots extracted from MD trajectories using molecular mechanics/generalized Born surface area (MM/GBSA) method.

## Results and discussions

**Fig. 2:**
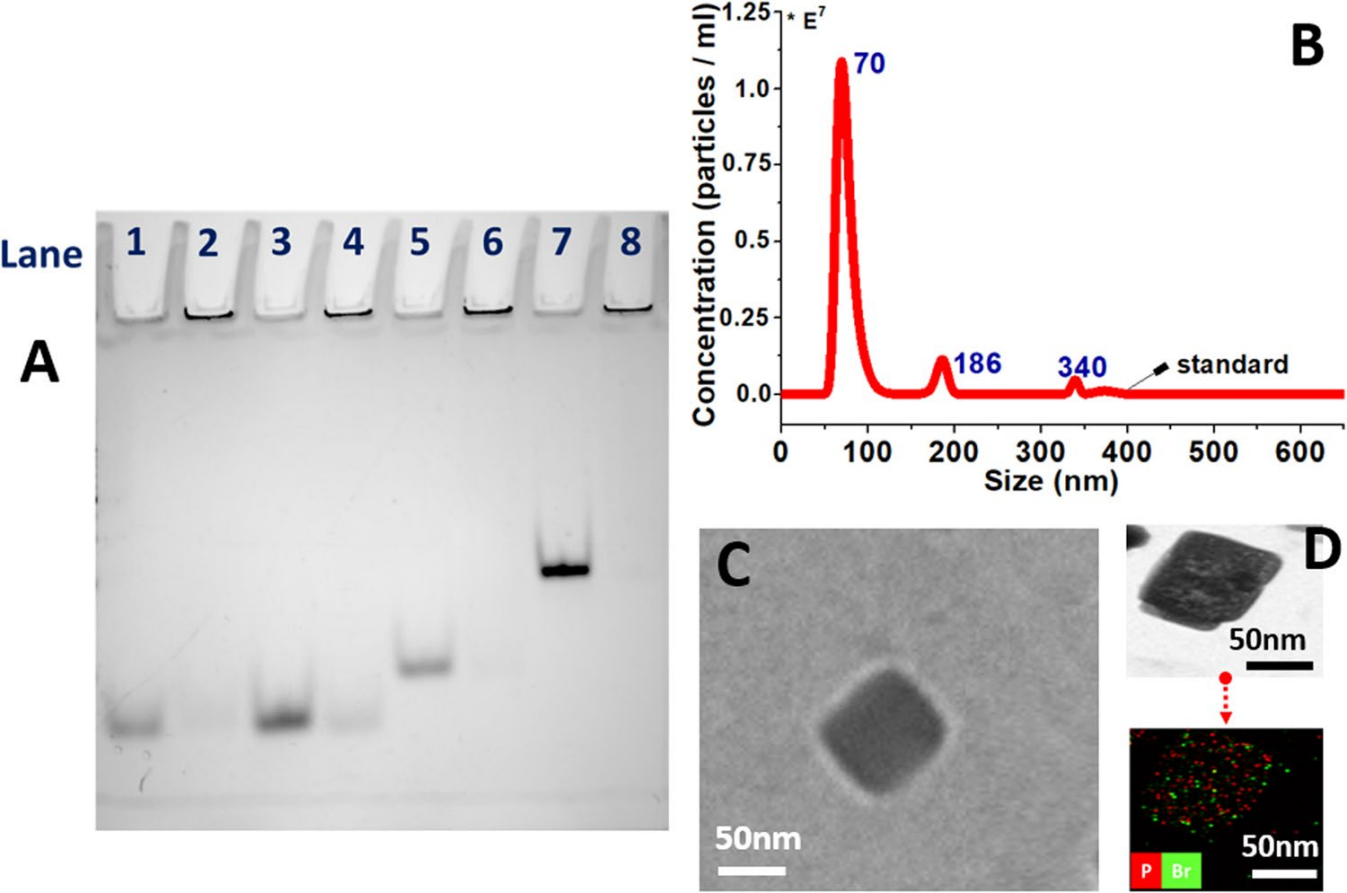
**A** Polyacrylamide gel electrophoresis (PAGE) of self-assemble nanoparticles (NPs-18nt, 36nt and 72nt) with ONs-18nt/36nt/72nt and C1/C2; Lane 1, corresponds to the 1 µM of ONs-18nt; Lane 2 corresponds to the mixed equivalent volume of ONs-18nt with C2; Lane 3 corresponds to the 2 µM of ONs-18nt, Lane 4 corresponds mixture of equivalent volume of ONs-18nt with C1; Lane 5, corresponds to the 1 µM of ONs-36nt; Lane 6 corresponds to the mixture of equivalent volume of ONs-36nt with C1; Lane 7 corresponds to the 1 µM of ONs-72nt, Lane 8 corresponds to the mixture of equivalent volume of ONs-72nt with C1; full version of the PAGE in the supporting information Figure S2A, Crosslink 19:1; **B** Nanoparticle tracking analysis (NTA) of NPs-18nt with ONs-18nt and C1; **C** Transmission electron microscopy (TEM) images of NPs-18nt with ONs-18nt and C1, and its **D** EDX mapping

The halogenated organic small molecules, including trans-1,4-dibromo-2-butene (denoted: C1), and 2-(4-bromobut-2-en-1-yl)isoindoline-1,3-dione (C2), are used as a proof of concepts. The key feature of these small molecules are comprised of allyl bromide scaffold with 6 to 16 atoms at a molecular mass of 213–282 g/mol. Small molecules are chemical chain structures that exhibited available H-bond acceptors, and contained chemical space for site-specific conjugation. The unique structure of small molecules triggered oligonucleotides (CTT GAG AAA GGG CTGCCA: ONs-18nt, CTT GAG AAA GGG CTGCCA CTT GAG AAA GGG CTGCCA: ONs-36nt, CTT GAG AAA GGG CTGCCA CTT GAG AAA GGG CTGCCA CTT GAG AAA GGG CTGCCA CTT GAG AAA GGG CTGCCA: 72nt, 5ʹ to 3ʹ) to fold into three-dimensional anionic nanoparticles (NPs). The NPs were assembled at higher concentrations of C1 compare to oligonucleotides (Fig. 2A, S1 and experimental procedure). With the increase in the length of oligonucleotides, the sizes of NPs are about ~ 70 nm (NPs-18nt), ~ 102 nm (NPs-36nt), and ~ 170 nm (NPs-72nt), respectively (Video S1 and Fig. 2B, S3 and S4). TEM images showed the morphology of the NPs, and the element analysis confirmed the coexistences of bromine (i.e., small molecules) and phosphorus (i.e., oligonucleotides) in the NPs (Fig. 2C, D and S5). Further, zeta potential shows -6.7 mV (NPs-18nt), -7.2 mV (NPs-36nt) and -6.0 mV (NPs-72nt).

The small molecules C1 modified by bicyclic hetero group i.e.; C2 (see synthetic procedure) which form NPs with different sizes of oligonucleotides (Fig. S1 and S6A-C). In contrast, small molecule N-(4-bromobutyl) phthalimide under similar conditions cannot be assembled. These comparable results show that the allyl bromide scaffold plays a key role in the formation of NPs. Subsequently, we investigated the interaction between the small molecules (C1 or C2) and differential poly nucleobases, such as adenine (A), cytosine (C) and thymine (T) (Table S1). Among them, A shows more favorable to form NPs than other nucleobases (Fig. S6D-E), suggesting the base-specificity in the interaction with the small molecules.

**Fig. 3:**
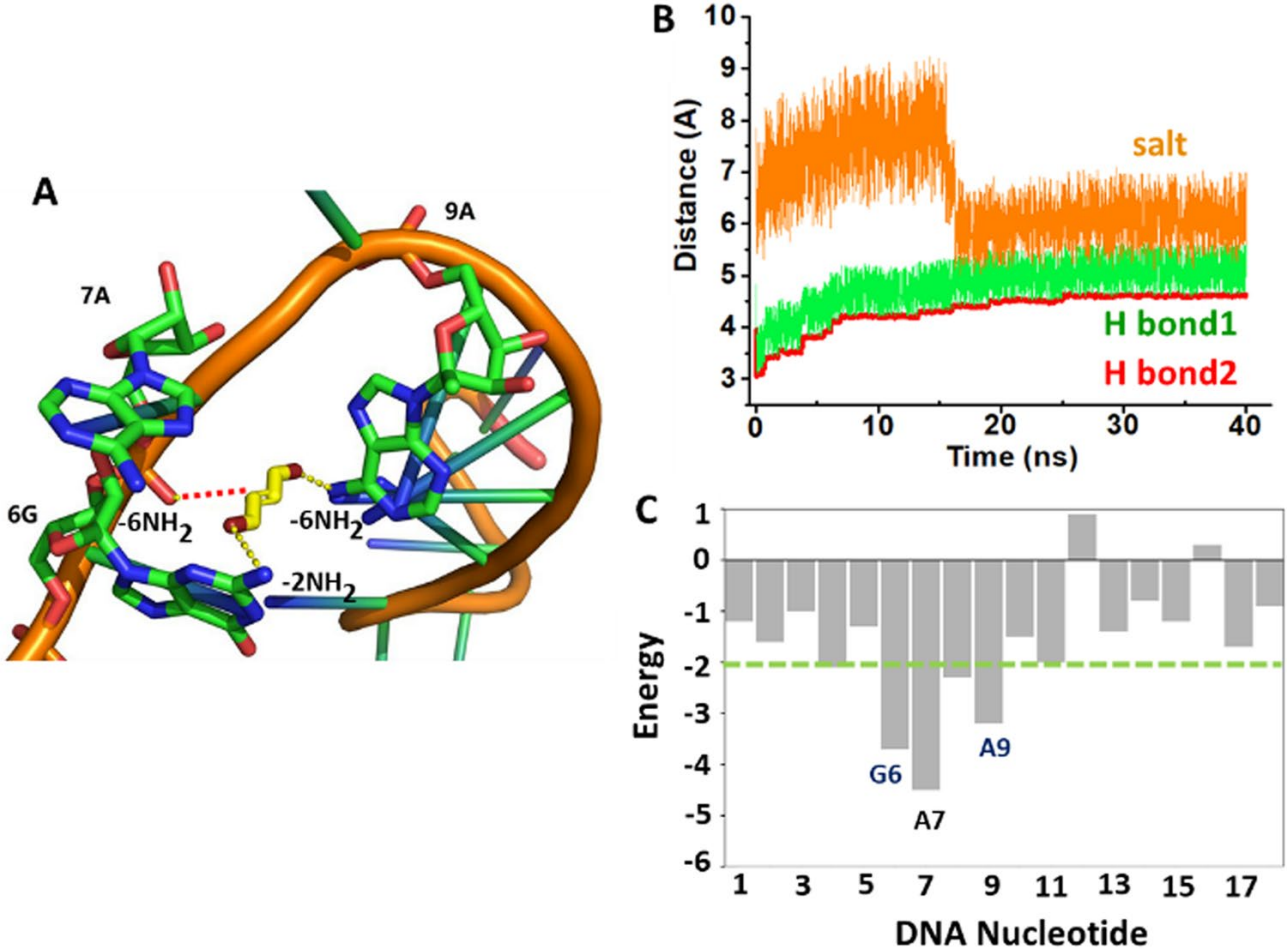
**A** The interaction patterns between ONs-18nt and C1 contribute to the stability of the complex, **B** The atomic distance between small molecules and nucleic bases as a function of simulation time, **C** Per nucleotide-based de-composition of the binding free energy of ONs-18nt and C1 complexes calculated by MM/GBSA

The molecular dynamics (MD) simulation illustrates the binding conformational space of oligonucleotides available for small molecule C1 (Fig. 3A). The allyl bromide (two Br atom in C1) forms a hydrogen bond with the -NH unit of 6-amine adenine and 2-amine guanine in the single-stranded oligonucleotides-18nt (5′-G6_A9_A7). The salt bridge interaction was found in distance 5–8 Å (Fig. 3B). The nucleotide-based decomposition energy lower than − 2.0 kJ/mol demonstrated the stability (Fig. 3C). The thermodynamic data exhibits that the assembly process is energetically favorable and driven by van der waal ( − 15.27 ± 0.39 kJ/mol), electrostatic interactions ( − 2.35 ± 0.09 kJ/mol) and binding energy ( − 18.95 ± 0.26 kJ/mol). On the other hand, C2 and oligonucleotides ONs-36nt simulation data support the interaction between 5′-A18 and allyl bromide units (Fig. S7). The assembly process is favorable and driven by van der waal ( − 13.25 ± 0.36 kJ/mol), electro-static interactions ( − 3.68 ± 0.49 kJ/mol), polar salvation (2.51 ± 0.24 kJ/mol), and non-polar salvation ( − 3.72 ± 0.19 kJ/mol), with the binding energy of  − 18.03 ± 0.92 kJ/mol.

## Conclusions

In conclusion, we have discovered halogenated allyl bromide derivative small molecules, capable of specifically interacting with polyanionic oligonucleotides to generate size-controllable self-assembled NPs. Our studies suggest a new kind of small molecule scaffolds for oligonucleotides intercalation to generate anionic small molecules. Further study is needed to explore the biomedical applications of the self-assembled oligonucleotide NPs.

## Supporting information available

Additional supplementary videos of ONs-18nt based NPs-18nt with C1, table, supporting figures and detailed of molecular dynamics simulations and spectral data of small molecules C2.

## Supplementary Information

Additional file1

Additional file2

## Data Availability

All the data generated or analyzed during this study are incorporated in this article and also its supplementary information files without restrictions.
