# Supplementary material for: Oligonucleotide nanoassemblies with allyl bromide scaffold-based small molecules
Source: Discov Nano. 2023 Jun 3;18(1):81. doi: 10.1186/s11671-023-03846-0 (PMC10239407; doi:10.1186/s11671-023-03846-0)
Supplement: Supplementary file 1 — Additional file1 [file 11671_2023_3846_MOESM1_ESM.docx]

**Oligonucleotide nanoassemblies with allyl bromide scaffold-based small molecules**

Sk Jahir Abbas^a*^, Sabina Yesmin^b^, Fangfang Xia^a^, Sk Imran Ali^c^, Zeyu Xiao^a,d*^, Weihong Tan^a,e,f*^

^a^ Institute of Molecular Medicine, Renji Hospital, Shanghai Jiao Tong University School of Medicine, Shanghai 200240, China. ^b^ Department of Physics, National Dong Hwa University, Hualien 97401, Taiwan. ^c^ Department of Chemistry, University of Kalyani, West Bengal, India. ^d^ Department of Pharmacology and Chemical Biology, Shanghai Jiao Tong University School of Medicine, Shanghai 200025, China. ^e^ Institute of Basic Medicine and Cancer (IBMC), Chinese Academy of Sciences, Hangzhou, Zhejiang 310022, China. ^f^ Molecular Science and Biomedicine Laboratory (MBL), Aptamer Engineering Center of Hunan Province, Hunan University, Changsha, Hunan 410082, China.

**Additional Supplementary Videos**

**Video S1: Oligonucleotides (18nt) based NPs with small molecules C1.**

**
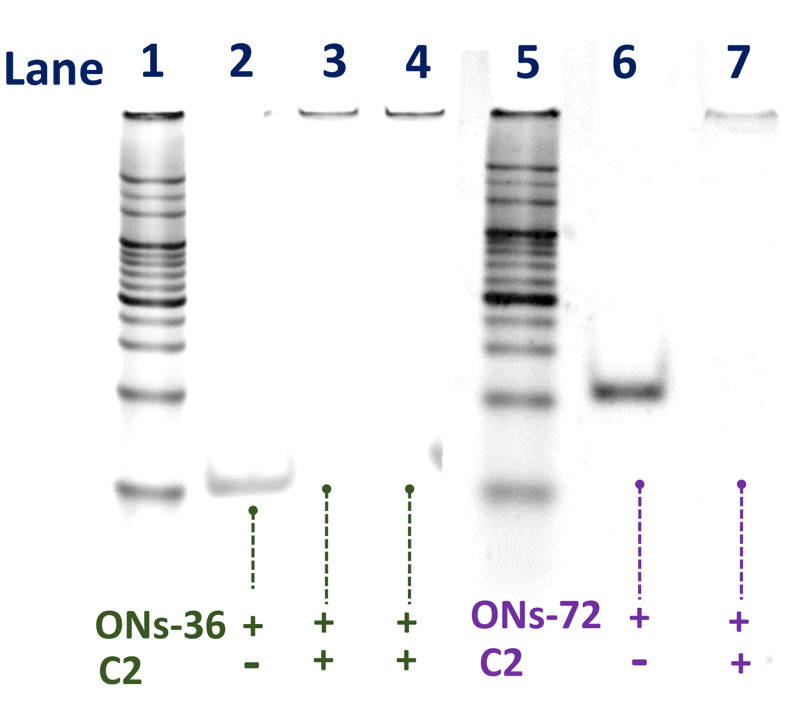
**

**Figure S1:** (A) Polyacrylamide gel electrophoresis (PAGE) of self-assemble nanoparticles (NPs). (B) Lane 1 and 5 DNA ladder marker, Lane 2, and 7 corresponds to the 1 and 1 µM of ONs-36nt and ONs-72nt respectively. Lane 3, 4 and 7 corresponds to the mixed equivalent volume of of ONs-36nt, and ONs-72nt with C2 respectively, lane 4 represent half of original concentration of C2. Full version of the PAGE in the Figure S2B and C.

**
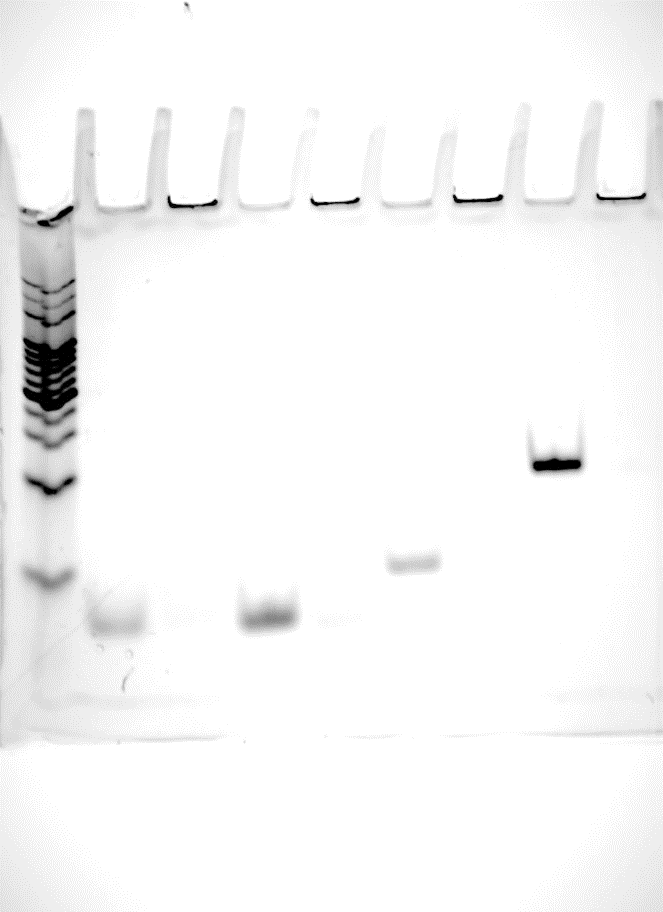

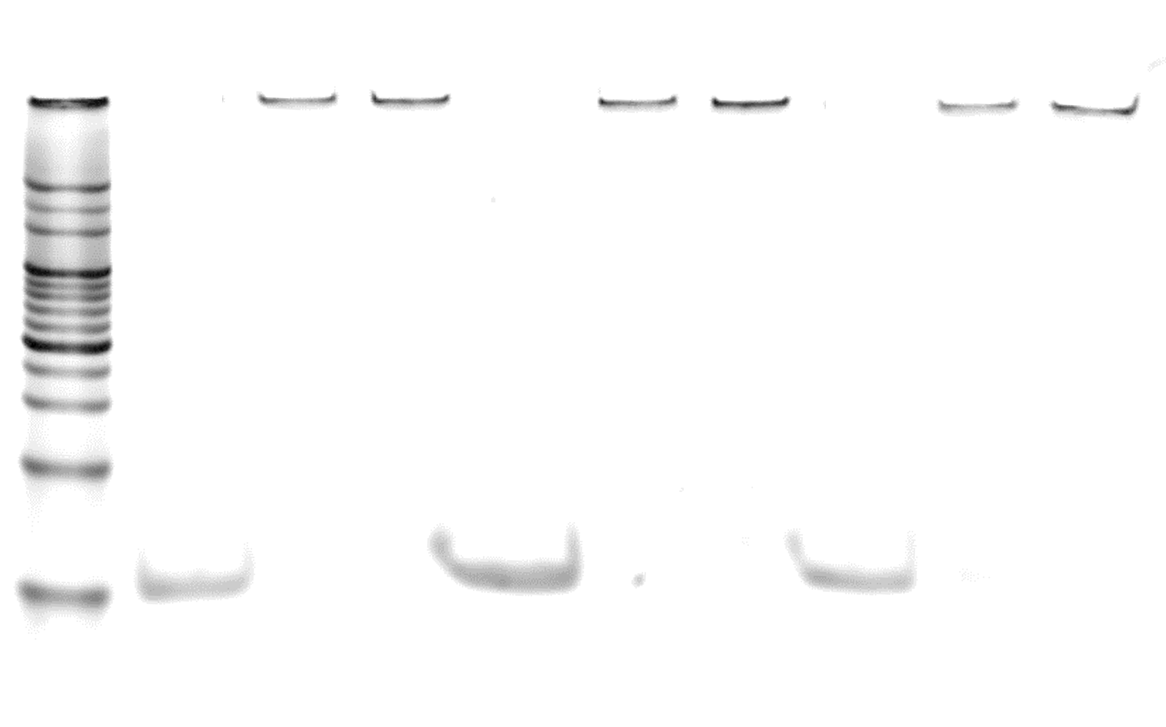
** **
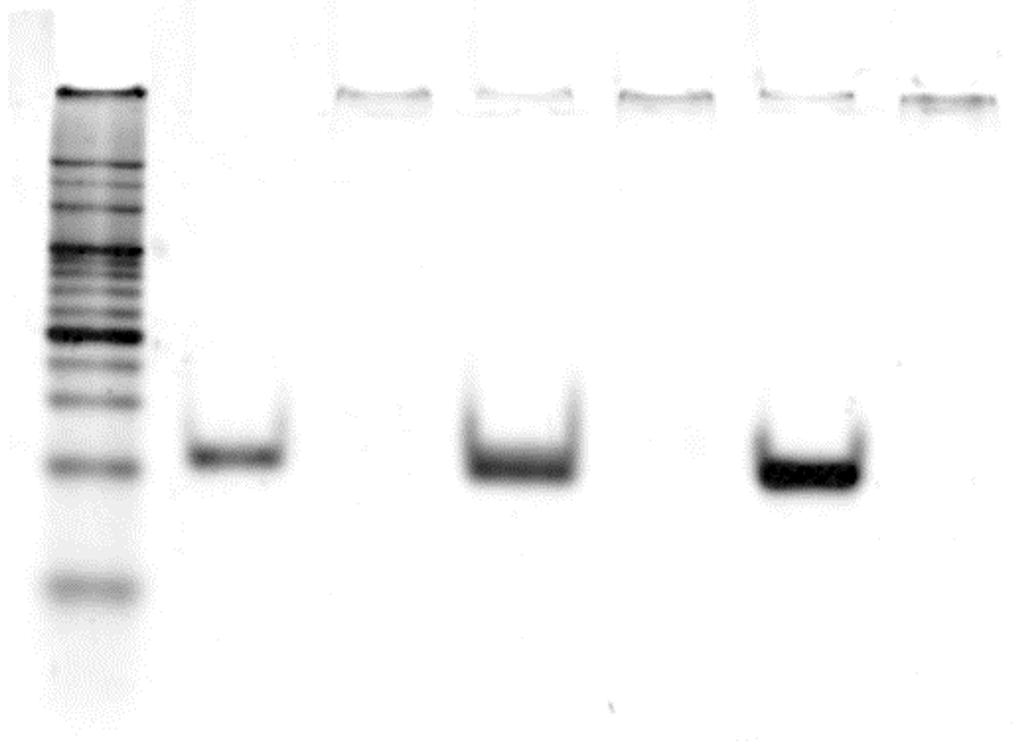
**

**B**

**A**

**C**

**Figure S2:** Polyacrylamide gel electrophoresis (PAGE) full version image of the Figure 1A and S2


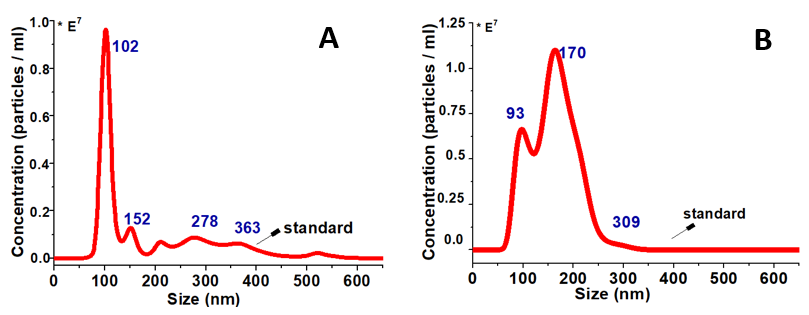


Figure S3: Nanoparticle tracking analysis (NTA) of ONs NPs of (A) NPs-36nt and (B) NPs -72nt with C1 respectively.


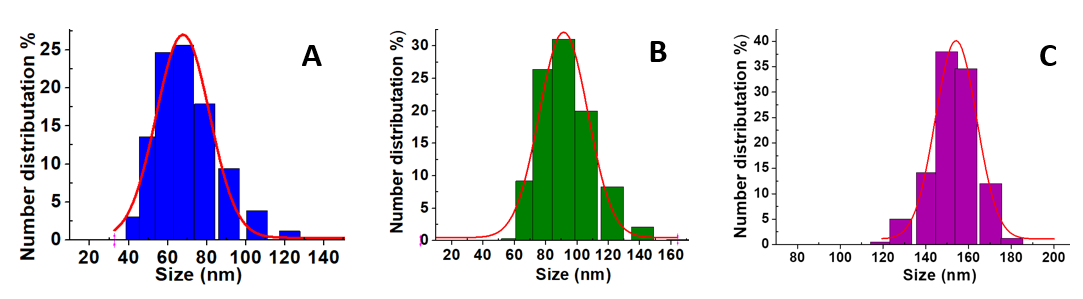


Figure S4: Dynamic light scattering (DLS) particle size distribution analysis of ONs NPs of (A) NPs-18nt, (B) NPs-36nt and (C) NPs-72nt with C1 respectively.


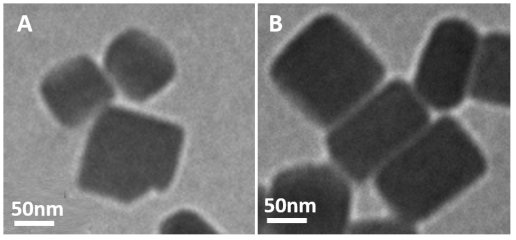


Figure S5: (A-B) Transmission Electron Microscopy (TEM) images of ONs NPs of 36nt and 72nt with C1 respectively.


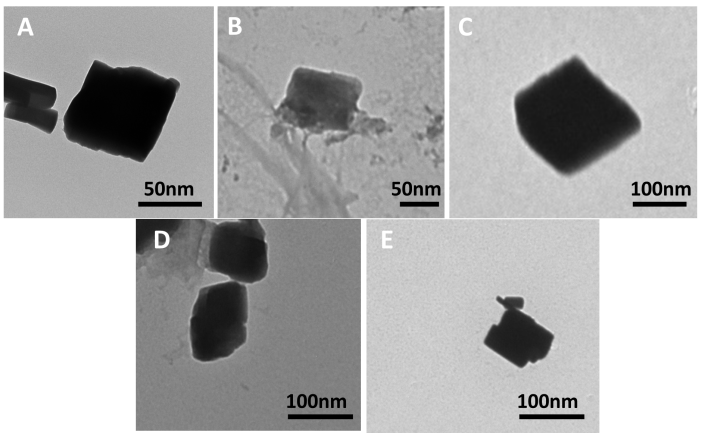


**Figure S6.** TEM images of ONs NPs of (A) 18nt, (B) 36nt, and (C) 72nt with C2 respectively; NPs-18nt of poly-A with (D) C1, and (E) C2.


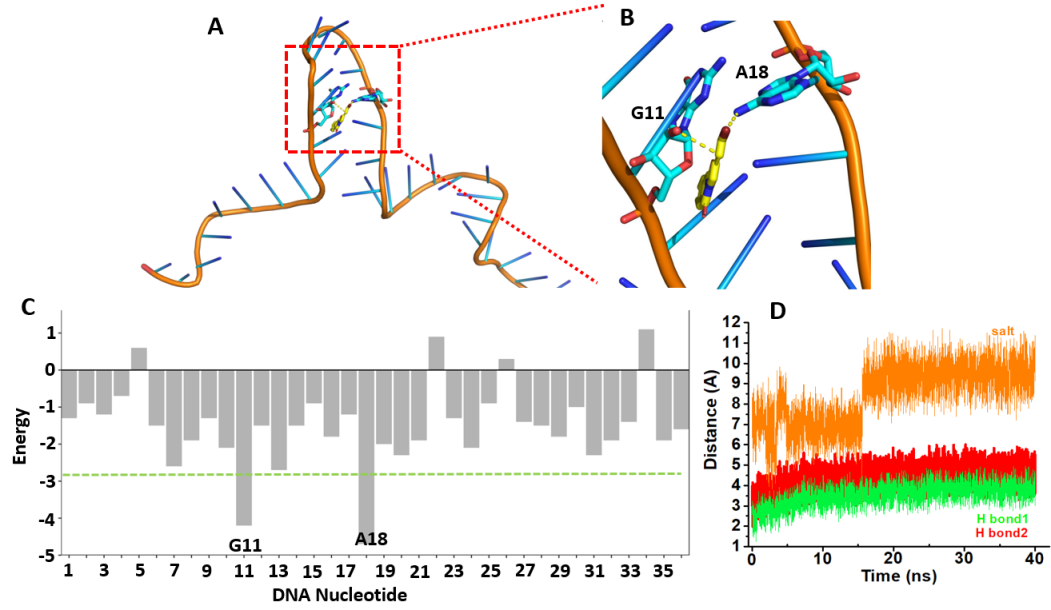


**Figure S7.** (A) Interaction patterns between ONs-36nt and C2, its expanded view (B), and (C) Per nucleotide-based decomposition of the binding free energy of ONs-36nt and C2 complexes calculated by MM/GBSA, (D) The atomic distance vs simulation time plot between C2 and ONs-36nt nucleic bases.

**Table S1.** The detailed information of oligonucleotides (ONs) sequences

| **Primer** | **(5' to 3')** |
| --- | --- |
| **Poly-A** | AAAAAAAAAAAAAAAAAAAAAAAAAAAAAAAAAAAA |
| **Poly-C** | CCCCCCCCCCCCCCCCCCCCCCCCCCCCCCCCCCCC |
| **Poly-T** | TTTTTTTTTTTTTTTTTTTTTTTTTTTTTTTTTTTT |

**General Information**

The HPLC purified synthesized oligonucleotides (shown in **Table 1b**) obtained Sangon Biotechnology Co., Ltd (Shanghai, China). All the organic chemicals and purified solvents were purchased from TCI Shanghai (China) and used without further purification. Purified water obtained from a Milli-Q water purification system (18.2 MΩ cm−1 resistivity, Millipore). ^1^H and ^13^C NMR spectra were recorded on a Bruker AvanceⅢ 400 and 100 MHz spectrometer (Germany) respectively. The UV-vis spectra were recorded with a SH-1000 Lab microplate reader (Corona Electric, Ibaraki, Japan). Gel electrophoresis imaged were detected using Bio-Rad ChemiDoc™ Touch Imaging System (USA). Transmission electron microscopy (TEM) images were recorded by Tecnai G20 microscope (USA) with voltage of 200 kV. Particle size and zeta potential were measured using DLS on a Zetasizer Nano particle analyser series (Malvern Instruments Ltd., England).


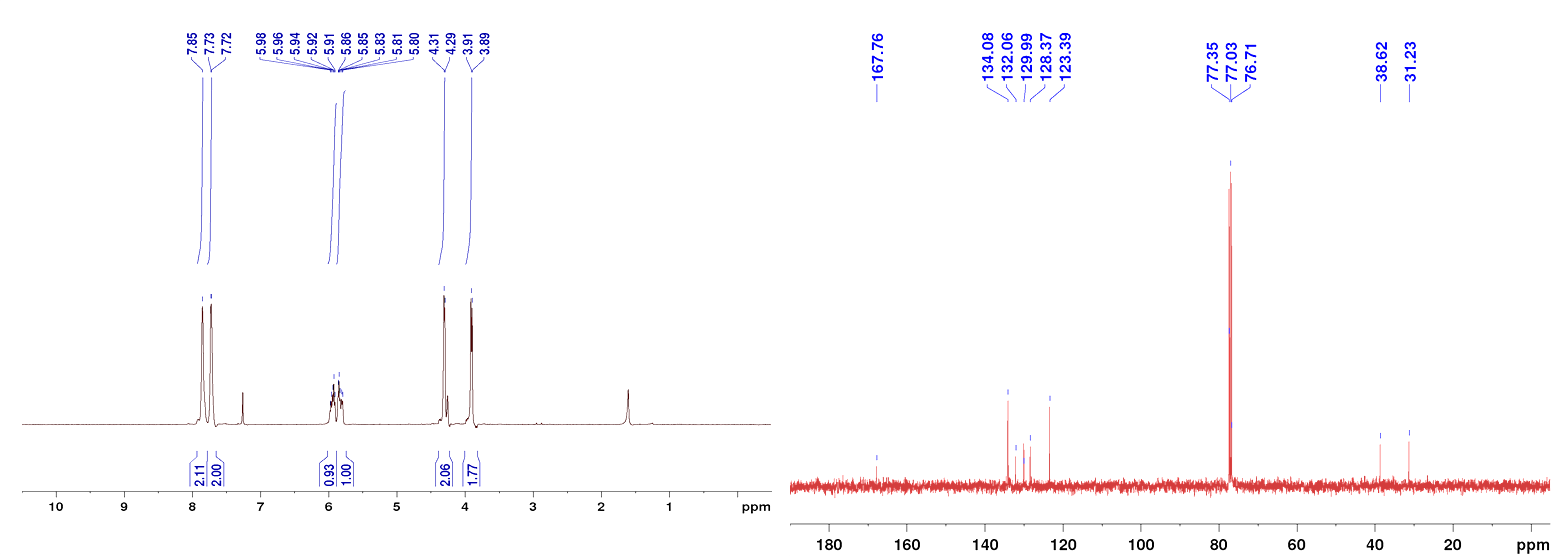


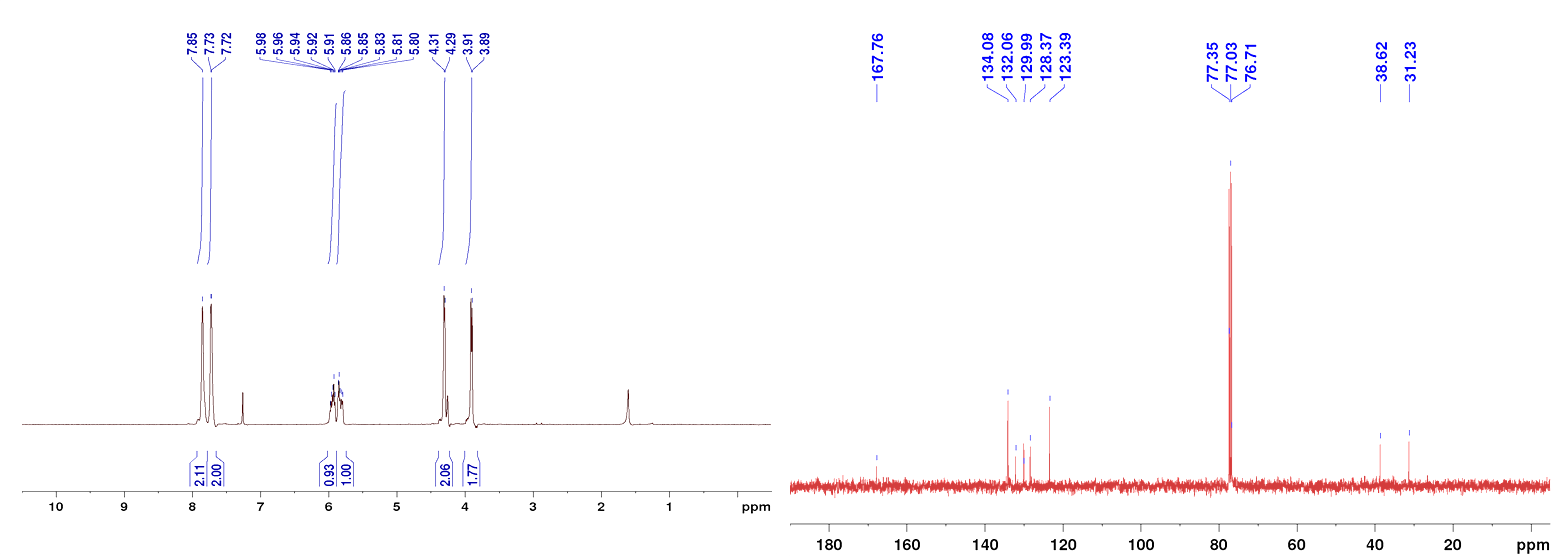


**NMR spectra of small molecules C2.** ^1^H and ^13^C NMR
